# Supplementary figures and images for: Compression-enabled interpretability of voxelwise encoding models
Source: PLoS Comput Biol. 2025 Feb 19;21(2):e1012822. doi: 10.1371/journal.pcbi.1012822 (PMC11867343; doi:10.1371/journal.pcbi.1012822)

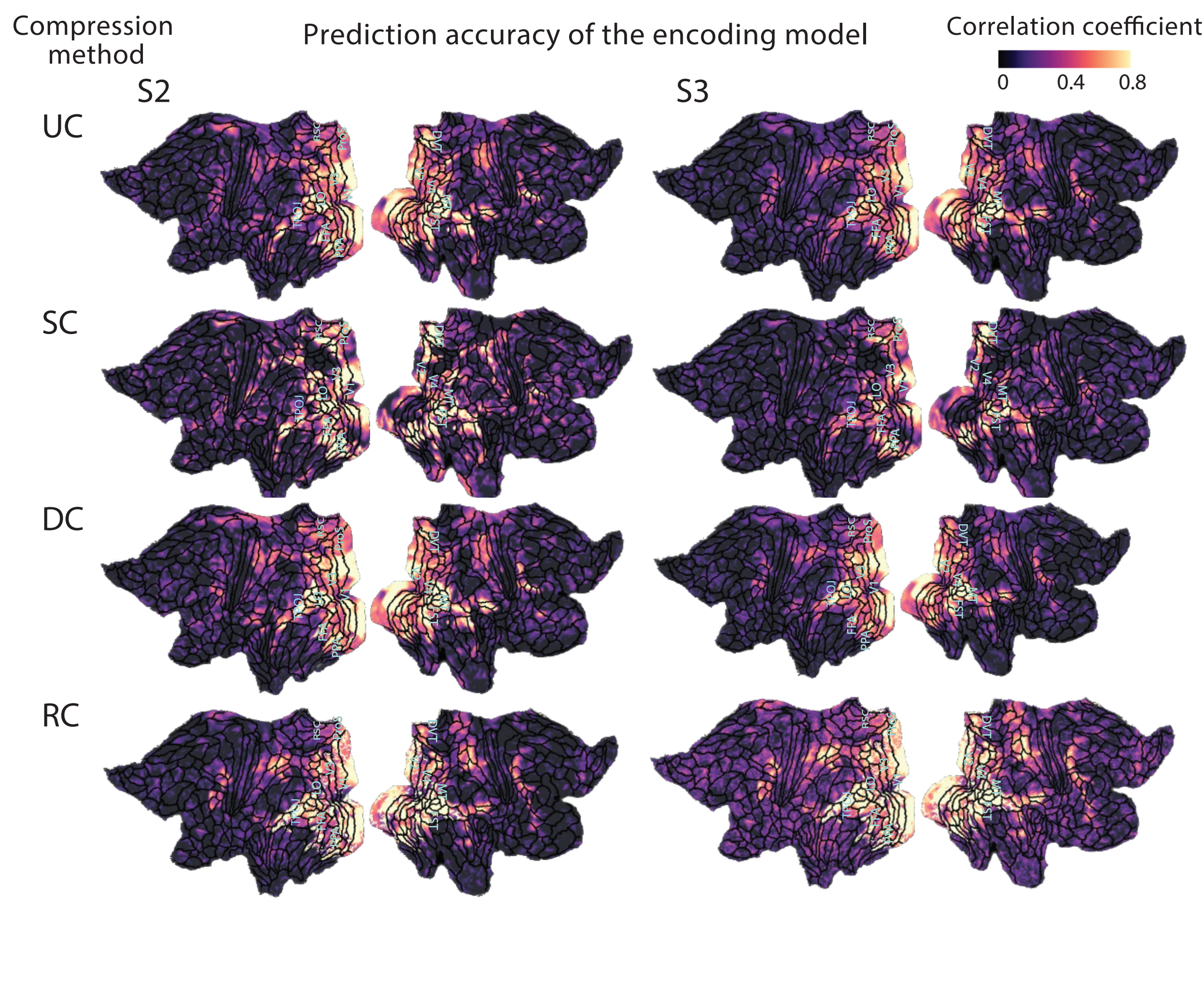

Supplement: S1 Fig — Compared to the uncompressed (UC) model, the structurally compressed (SC) model better estimates fMRI responses in ProS, DVT, and the lateral part of V1. The deep-compressed (DC) model is more accurate in the central part of V1, V2, V3, and the lateral part of V4. The receptive field-compressed (RC) model better estimates the lateral part of V1, V2, V3, V4, as well as the TPOJ and FST areas. (PDF) [file pcbi.1012822.s001.pdf]

# Difference between compressed and uncompressed models

Correlation coefficient

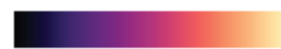

0

0.2

SC

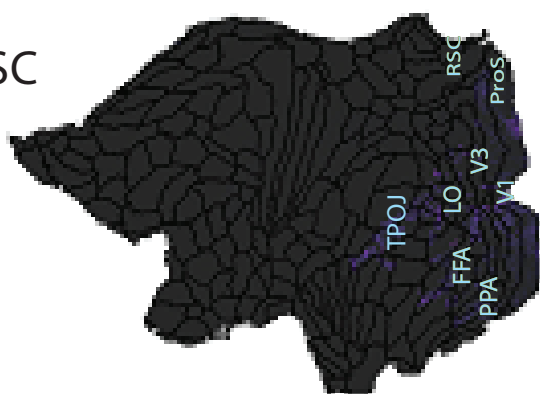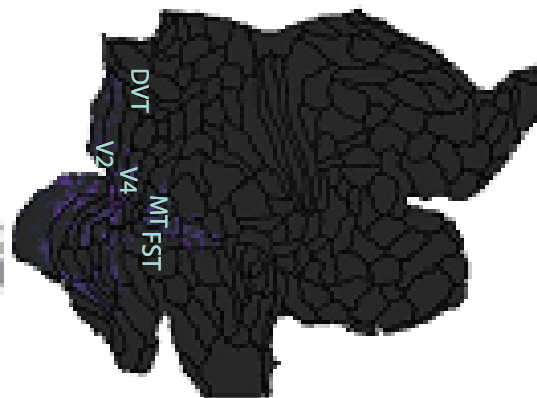

DC

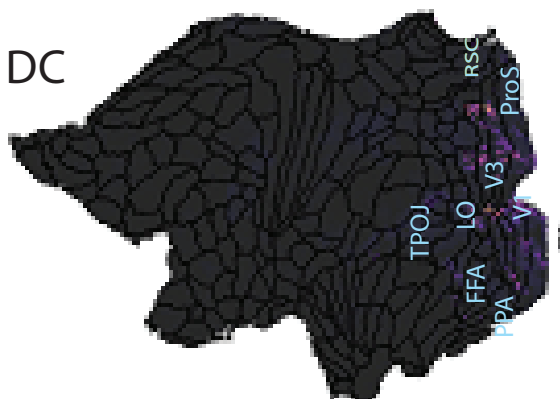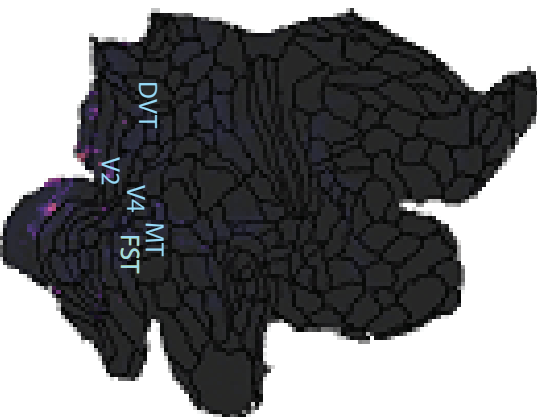

RC

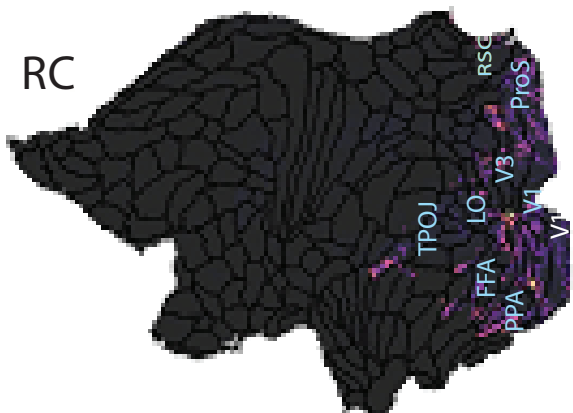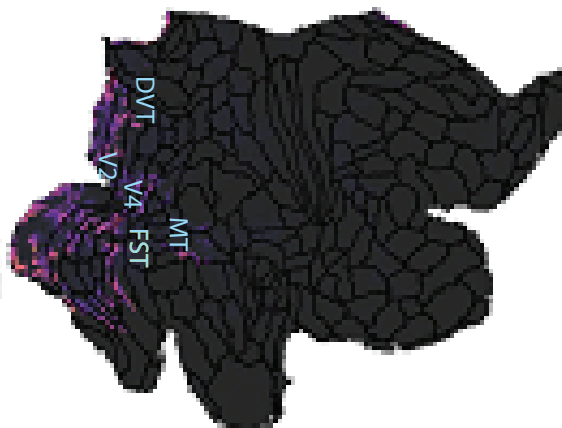

Supplement: S2 Fig — (PDF) [file pcbi.1012822.s002.pdf]

# Comparison of compressed models and uncompressed model

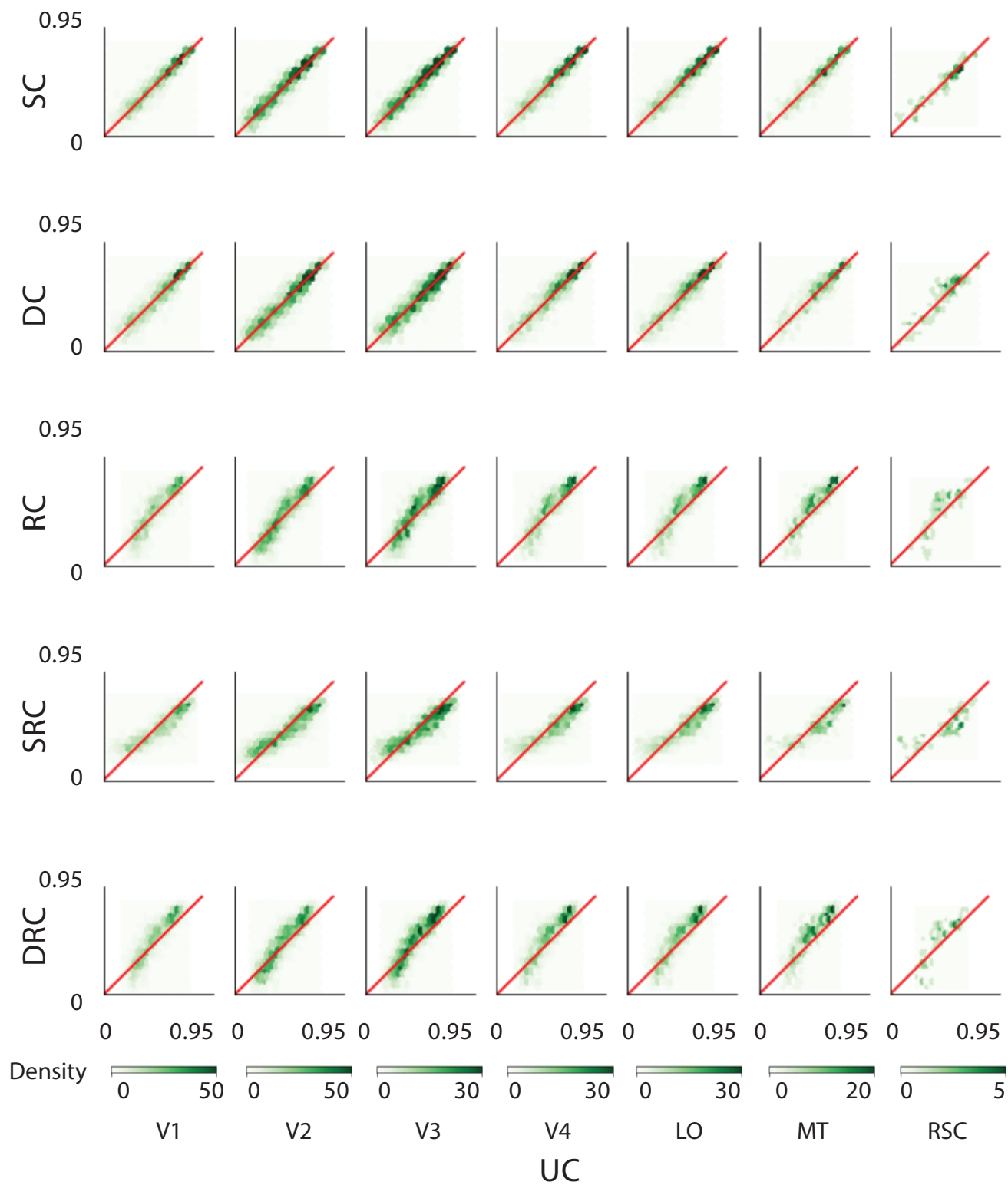

Supplement: S3 Fig — Each dot corresponds to one voxel. Columns represent different visual areas. Rows represent different compression techniques. (PDF) [file pcbi.1012822.s003.pdf]

Comparison of compressed models and uncompressed model

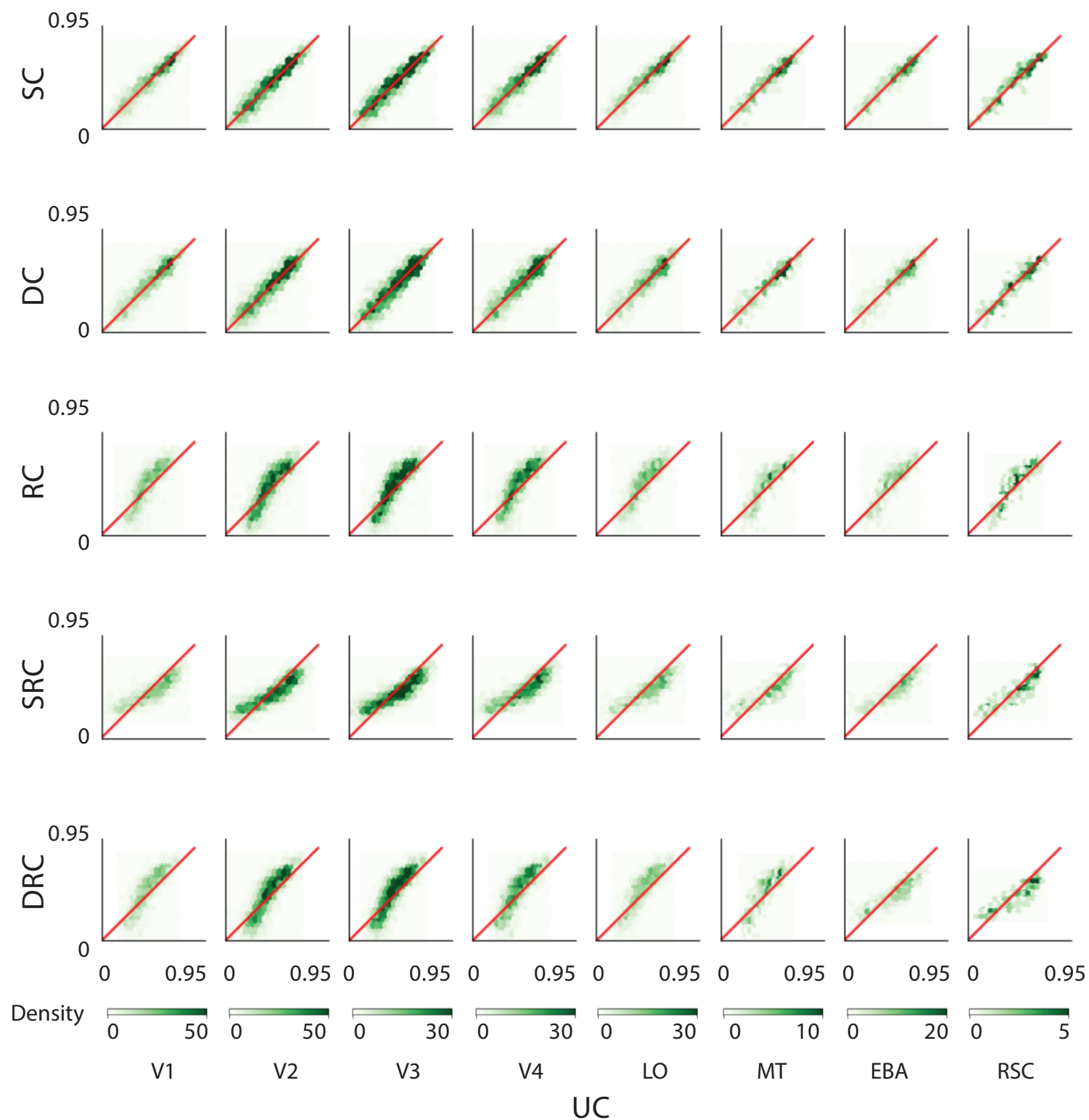

Supplement: S4 Fig — Each dot corresponds to one voxel. Columns represent different visual areas. Rows represent different compression techniques. (PDF) [file pcbi.1012822.s004.pdf]

# Comparison of compressed models and uncompressed model

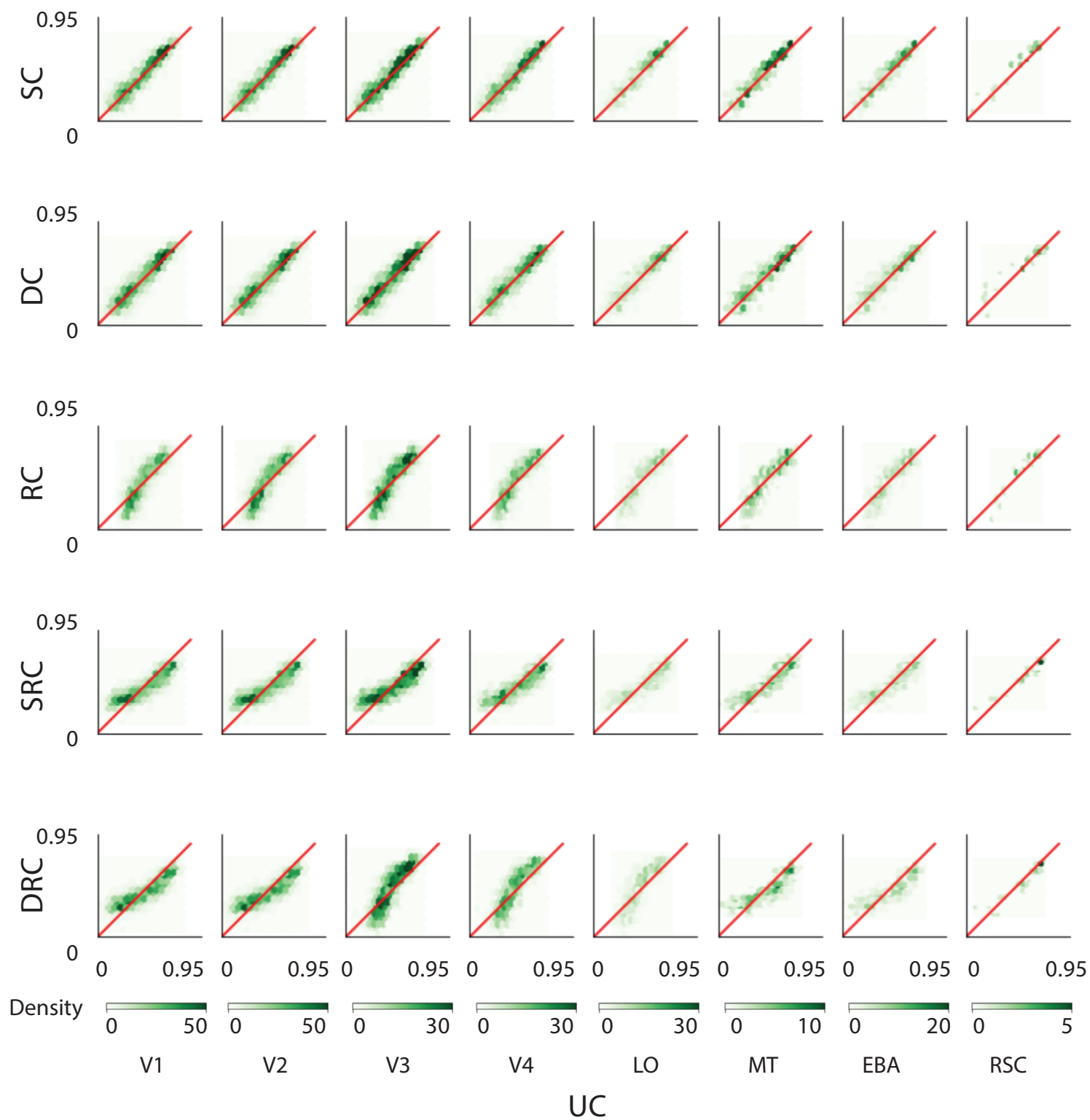

Supplement: S5 Fig — Each dot corresponds to one voxel. Columns represent different visual areas. Rows represent different compression techniques. (PDF) [file pcbi.1012822.s005.pdf]

Comparison of compressed models and uncompressed model

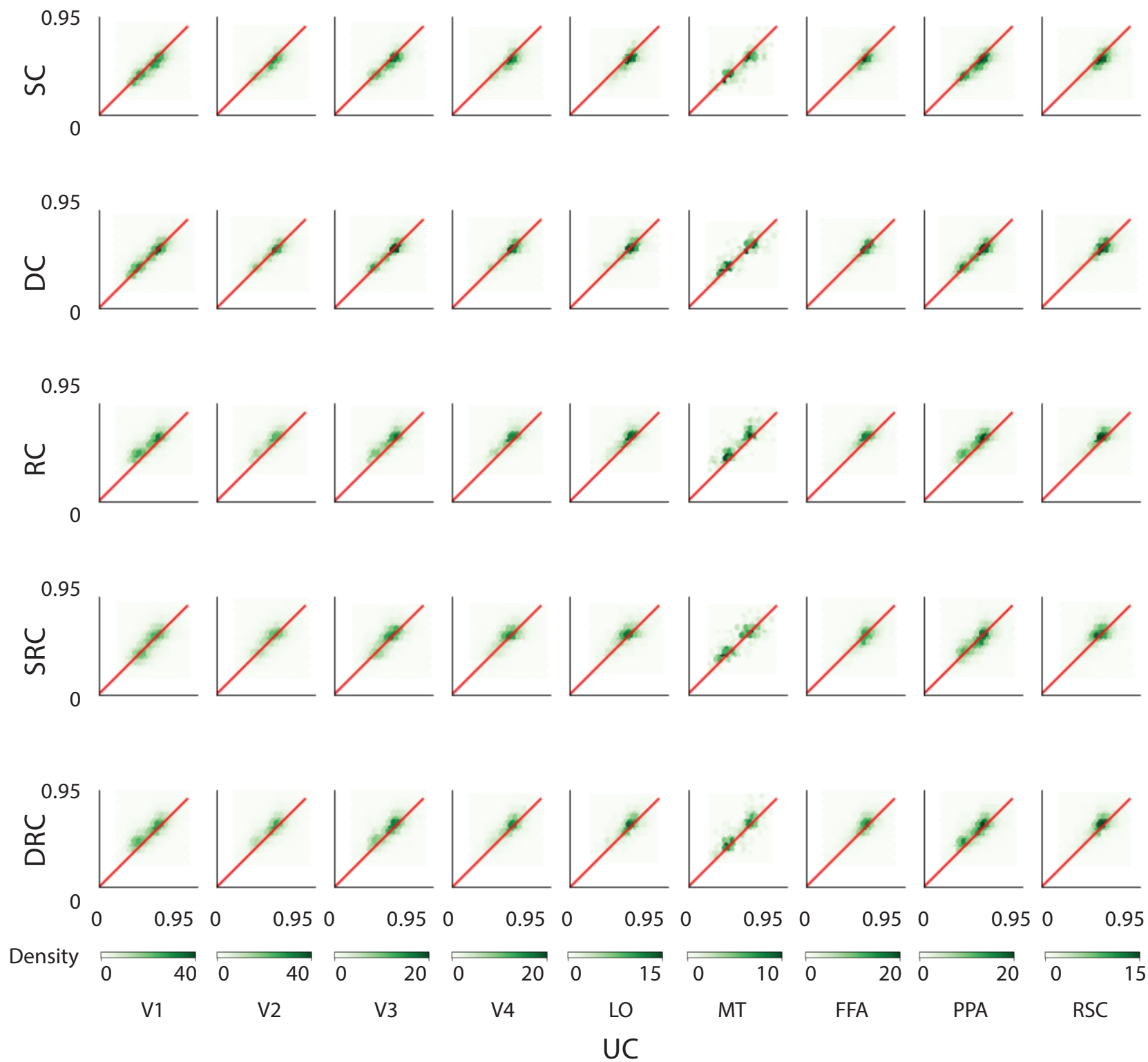

Supplement: S6 Fig — Each dot corresponds to one voxel. Columns represent different visual areas. Rows represent different compression techniques. (PDF) [file pcbi.1012822.s006.pdf]

Comparison of compressed models and uncompressed model

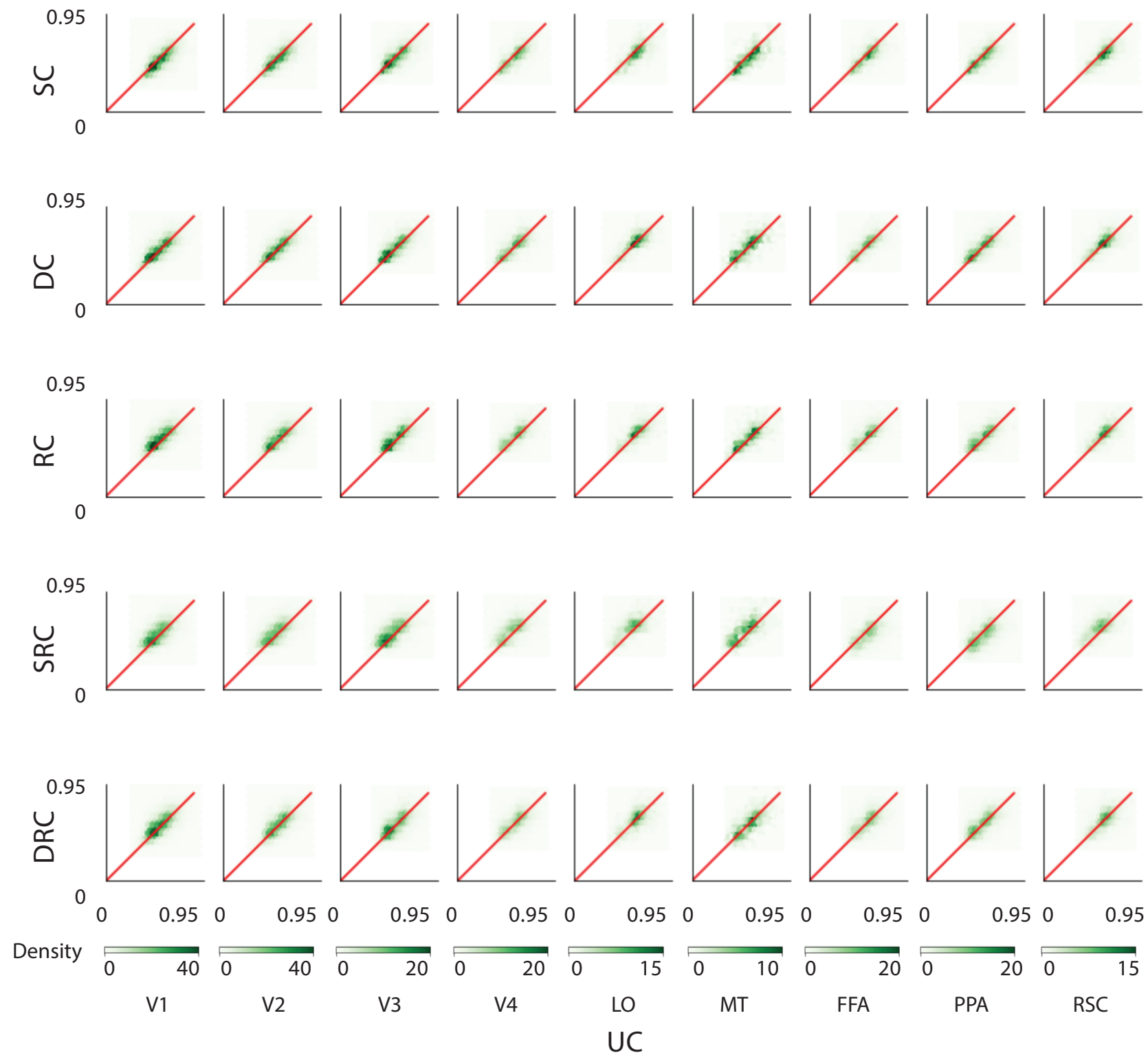

Supplement: S7 Fig — Each dot corresponds to one voxel. Columns represent different visual areas. Rows represent different compression techniques. (PDF) [file pcbi.1012822.s007.pdf]

Comparison of compressed models and uncompressed model

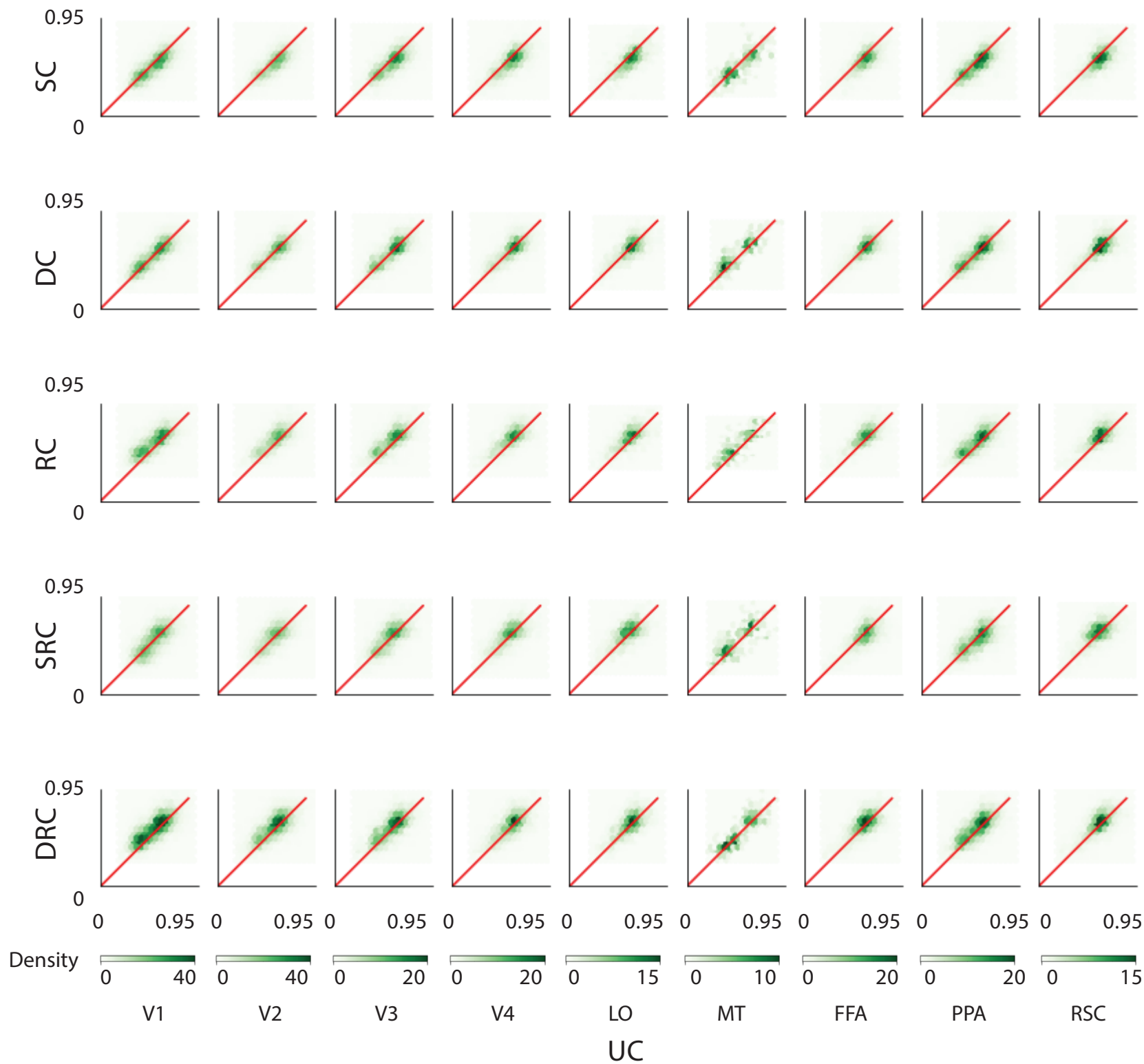

Supplement: S8 Fig — Each dot corresponds to one voxel. Columns represent different visual areas. Rows represent different compression techniques. (PDF) [file pcbi.1012822.s008.pdf]
